# User-Centered Research on Breast Cancer Patient Needs and Preferences of an Internet-Based Clinical Trial Matching System

**DOI:** 10.2196/jmir.9.2.e13

**Published:** 2007-05-15

**Authors:** Nancy L Atkinson, Holly A Massett, Christy Mylks, Bethany Hanna, Mary Jo Deering, Bradford W Hesse

**Affiliations:** ^5^Health Communications and Informatics Research BranchNational Cancer InstituteNIHRockvilleMDUSA; ^4^NCI Center for BioinformaticsNational Cancer InstituteNIHRockvilleMDUSA; ^3^School Nutrition AssociationAlexandriaVAUSA; ^2^Operations Research OfficeNational Cancer InstituteNIHRockvilleMDUSA; ^1^Department of Public and Community HealthUniversity of MarylandCollege ParkMDUSA

**Keywords:** Software design, user-computer interface, breast neoplasms, personal health records, qualitative research, user-centered design, human-computer interaction

## Abstract

**Background:**

Internet-based clinical trial matching systems have the potential to streamline the search process for women with breast cancer seeking alternative treatments. A prototype system was developed to leverage the capabilities of a personal health record system for the purpose of identifying clinical trials.

**Objective:**

This study examines how breast cancer patients perceive and interact with a preliminary version of an Internet-based clinical trial matching system, while taking into account the demands of diagnosis and treatment decision making.

**Methods:**

Breast cancer patients participated in small group discussions and interacted with the prototype website in a two-phase qualitative research process. The first phase explored the experience of breast cancer patients (n = 8) with treatment decision making, initial responses to the idea of Internet-based clinical trial matching systems, and reactions to the prototype site. In the second phase, a different set of breast cancer patients (n = 7) reviewed revised website content and presentation and participated in a usability test in which they registered on the system and completed a personal health record to set up the matching process.

**Results:**

Participants were initially skeptical of the prototype system because it emphasized registration, had a complicated registration process, and asked for complex medical information. Changing content and attending to usability guidelines improved the experience for women in the second phase of the research and enabled the identification of functionality and content issues, such as lack of clear information and directions on how to use the system.

**Conclusions:**

This study showed that women felt favorably about the idea of using the Internet to search for clinical trials but that such a system needed to meet their expectations for credibility and privacy and be sensitive to their situation. Developers can meet these expectations by conforming to established usability guidelines and testing improvements with breast cancer patients. Future research is needed to verify these findings and to continue to improve systems of this nature.

## Introduction

 Despite efforts to improve recruitment to cancer clinical trials, cancer patients continue to have difficulty understanding the research process and finding trials that they are interested in and qualify for [1]. Communications technology may be able to facilitate the clinical trial search process [2,3]. Already, the Internet provides an accepted tool for health information seeking [4] and clinical trial enrollment [3], and its use continues to spread widely throughout the United States [5]. Electronic personal health record (PHR) systems, another emerging technology, have the potential to further empower patients by enabling them to collect, store, and share their personal health information [6]. This paper explores the efficacy of enhancing an Internet-based prototype clinical trial matching system with the types of information collected in electronic PHRs to help women with breast cancer locate clinical trials most appropriate for their health condition.

Clinical trial enrollment is necessary to conduct the trials that advance medical research and practice [7]. Patients make the decision to participate in clinical trials for a variety of reasons, including the desire to help in the development of new and improved medical treatments [8]. Some participate out of a sense of altruism and wanting to help others who may be coping with a particular health problem [9,10]. Other patients seek trials that provide access to promising new treatments, which is particularly important if standard treatments have proven ineffective for them [10].

Nonparticipation in clinical trials is well documented. Many people are simply unaware of them or are uncomfortable with experimentation or participating in a trial with unknown outcomes; others fear losing health insurance or control of their treatment [9-12]. The clinical trial search process serves as another barrier [9-11]. In order to find trials, patients must be versed in the medical terminology related to cancer diagnosis and treatment and must understand the clinical trial research process in general and the demands of trials [11]. For example, patients who do not understand randomization or the different phases of clinical trial research will have trouble identifying a trial they might prefer or even asking questions about the trial [11]. Finally, the nature of the trial protocol is a barrier to enrollment [10,11]. Patients must meet certain inclusion and exclusion criteria in order to qualify for a trial; specifically, they must match the profile of a certain type of patient for whom the treatment is intended. As a result, patients have often been frustrated in their attempts to identify, qualify for, and join a trial [13].

National efforts are being made to empower patients through access and control of their medical records by maintaining a PHR [6]. The PHR is “an electronic application through which individuals can access, manage, and share their health information in a secure and confidential environment” [14]. PHRs have the potential to place the same information that clinicians have in the hands of patients so they can make informed decisions about their health care. A recent survey showed that the general public perceives that PHRs would improve their understanding of health conditions and their sense of control [15]. For the purpose of finding clinical trials, a detailed PHR-modeled system may also have the potential to facilitate a productive search by matching specific medical history information to the inclusion and exclusion criteria of relevant trials [3].

The purpose of the current study was to assess the usefulness, appeal, and functionality of an Internet-based clinical trial matching program from the perspective of the intended users: breast cancer patients. BreastCancerTrials.org (BCT) was the prototype system used in the current research study. This Internet-based system was initiated by two breast cancer survivors, working with the University of California, San Francisco (UCSF) Comprehensive Cancer Center and the Center of Excellence for Breast Cancer Care. BCT was subsequently developed as a working tool by the Center for Bioinformatics at the National Cancer Institute (NCI), in collaboration with UCSF and NCI’s Office of Communications, which manages NCI’s clinical trials database, PDQ. The site was designed to match patient medical histories to the eligibility criteria of participating clinical trials via a patient-created online medical record similar to a PHR that captures detailed information about current health, diagnosis, and treatment. Unlike most clinical trial search tools, BCT narrows the list of clinical trials based on the person’s actual medical record and seeks to limit frustration by improving the chance of a productive search.

Most services to search clinical trials provide listings of trials that may or may not allow users to filter results. More elaborate decision support tools such as BCT [16,17] have been developed for clinicians and investigators to aid in identifying potential research participants. A previous study on one Internet-based matching system for patients [3] showed that people were willing to use such a system and were able to enroll in trials. However, the findings were based on the people who actually found a match rather than all the users of the system [3], so the study did not reveal the reactions of users overall or any issues related to the experience of using the system.

Little other research has been conducted on Web-based search tools for cancer clinical trials. One study looked at cancer clinical trial websites that could be located from popular Internet search engines [18] and found that almost half of the 66 sites that were identified offered some kind of clinical trial search function. Researchers concluded that clinical trial websites had promise for increasing clinical trial recruitment, but barriers still existed that would hinder their use, such as large amounts of complex information and the lack of confidentiality. These findings were confirmed in a study [19] of the clinical trial search tools on the websites of NCI Comprehensive Cancer Centers. Most search mechanisms allowed the user to search by type of cancer but used medical terminology rather than lay language (eg, melanoma instead of skin cancer), and search results were found to be written at an eleventh grade level according to a readability analysis.

Because using BCT depends on having patients register and accurately complete the entire medical record for successful matching to clinical trials, research was necessary to assess how patients perceived, interacted with, and understood the site. This research also sought to establish how such a system fits into the experience of breast cancer patients when making treatment decisions and to assess whether use of the system posed an additional barrier [20]. Since many breast cancer patients are likely to be older, and older adults’ ability to use the Internet differs from other populations, the research also needed to examine interface design issues that may arise due to the demographics of the target population [21]. Three research questions guided the study:

1. How do breast cancer patients make treatment decisions in general and when presented with the prospect of an Internet-based clinical trial matching system?
2. What is the perceived usefulness of a prototype online system (ie, BCT) for matching breast cancer patients to clinical trials?
3. In what ways do the system’s design and functionality help or hinder the clinical trial matching process?

## Methods

### Research Design

This qualitative study was conducted in March 2005 in two phases. In the first phase, researchers focused on general breast cancer diagnosis and treatment experiences, reactions to clinical trial participation, and reactions to the general idea of an Internet-based clinical trial matching system and to the prototype system. Based on the results of the first phase, the introductory site content and approach (home page, etc) were revised (see the Multimedia Appendix for before and after versions of the home page). The second phase of the study was a usability test of the site that focused on participant reactions to the revised introductory materials and to their experience of completing a PHR and finding clinical trials.

The use of two phases allowed thorough discussion and exploration of the issues and system while also not overburdening the respondents. This strategy also allowed the researchers to assess whether the revised content was more effective than the original version in orienting users to the prototype system, which is consistent with the usability guideline of evaluating websites before and after making changes [20].

A key component of the research design was the use of triads, in which three women participated in group discussion with a moderator. Like focus groups, triads provide an opportunity to explore issues in depth but allow each person to convey more information in a session [22]. The smaller group size, however, enabled the researchers to provide a more intimate environment for the participants who were coping with a sensitive health problem.

### Sample

For the purposes of this research, NCI hired a professional recruitment firm to identify women eligible for the study from a marketing list they compiled on regular basis. The firm was provided a screener questionnaire to recruit a diverse mix of participants on the following criteria. All the participants in the study were female breast cancer patients age 18 or older living in the Washington, DC, metropolitan area. The study also sought to recruit patients who represented the full range of breast cancer diagnostic stages (stages 1 to 4) and patients with and without experience in clinical trials. Ethnic/racial diversity was requested but not required.

Women were excluded from participating in the study if they were employed by the US Department of Health and Human Services, the American Cancer Society, or other breast cancer foundations. Medical professionals, Web developers, information architects, and computer programmers were also excluded from participation because their technical knowledge and work experiences could influence their responses and perceptions. Women who did not use the Internet at least monthly were also excluded.

The first phase of the study included three triads of patients with stage 1 to 3 breast cancer who had not been in a clinical trial and one triad of women with stage 4 breast cancer who were currently in a clinical trial. The second phase consisted of one in-depth interview with a clinical trial patient with stage 4 breast cancer and two triads of patients with stage 1 to 3 breast cancer who had not been in a clinical trial. None of the participants from the first phase of the study participated in the second phase.

### Instrumentation

Both study phases covered four topics: (1) personal experience with breast cancer, (2) perceptions of clinical trials, (3) the process of finding out about clinical trials, and (4) reaction to the prototype website. The questions in the first phase focused more heavily on the first three topics, and the second phase focused on topics 3 and 4 (see Table 1 for specific question areas). Phase 1 participants were asked more extensively about their experiences with breast cancer diagnosis, treatment, and information seeking and about their clinical trial perceptions, while phase 2 participants were asked to share primarily what they experienced at the time of diagnosis and any experience they had with clinical trials. Phase 2 participants spent more time reviewing the revisions that had been recommended in phase 1. Phase 1 participants did not examine the prototype in depth after completing the registration process, but phase 2 participants were asked to complete a medical record and try to find a trial.

**Table 1:** Discussion topics by research phase and category

**Category**	Phase 1 Topics	Phase 2 Topics
Personal Experience	Breast cancer diagnosis Finding out about treatment options Ease or difficulty of finding information Physician’s role in treatment	Breast cancer diagnosis
Perceptions of Clinical Trials	Knowledge of clinical trials Positive and negative perceptions Finding out about clinical trials Perceptions of clinical trial participants Personal experiences in clinical trials	Personal experiences in clinical trials
Exploring Clinical Trials	How they would learn about clinical trials Knowledge and perceptions of online clinical trial tools	Personal experiences of learning about clinical trials, if any
Review of Prototype	Home page general impressions and acceptability Ease of navigation Review of background material provided on the site Review of privacy policy text General impressions and acceptability of information requested when completing the PHR/profile	Review of revised home page text, graphics, and layout Observation of initial expectations and reactions to site Ease of log-in process and perceived acceptance Ease of the PHR process and perceived acceptance Home page general impressions and acceptability Ease of navigation Reactions to logging in to the site Review of background material provided on the site Review of privacy policy and informed consent text General impressions and acceptability of information requested when completing the PHR/profile

### Data Collection

The study took place in the usability testing facility of the User-Centered Informatics Research Laboratory in NCI’s Operations Research Office. In both phases of the study, a skilled moderator not involved with website development conducted the interviews with the assistance of a usability specialist. Participants each sat at a computer with access to the BCT site so as to provide a real-time assessment of ease of use, desirability, and thought process. The iterative process first involved a group discussion of the first three topic areas, followed by a series of one-on-one computer interaction sessions with different parts of the website (eg, homepage, “About Clinical Trials” page), and then followed by group discussions reacting to each website part. Participants also were asked to provide written feedback on printed copies of the Web pages, making notes and highlighting sections they liked and disliked. These sessions were audiotaped and videotaped. Also, through a two-way mirror, research assistants observed the interviews, the women’s use of the prototype system, and their nonverbal reactions to working with it.

As described above, each phase had different goals, which influenced the data collection process. The first phase assessed overall clinical trial attitudes and experiences as well as reactions to the existing site and introductory materials on the home page and subpages. After phase 1 was completed, a website designer revised the BCT site pages based on the feedback obtained and mocked up paper drafts of each revised page. During phase 2, participants took part in a usability test of the existing website in which they were asked to attempt to accomplish three tasks: register, fill in the medical record forms, and review matches. For each task, they were first observed using the site and were then given the opportunity to discuss their experiences as a group. Following an initial review of the site, participants reviewed the revised version and were asked to react as a group to the changes.

This study was determined to be exempted research because it was designed to evaluate consumer satisfaction for quality improvement rather than individual behavior. Nevertheless, only participants who agreed to participate and signed informed consent forms were included in the study. The data were anonymous and reported in the aggregate with no identifiers.

### Data Analysis

The data were analyzed using content analysis, a qualitative method, via code mapping of transcripts [23]. Certain words, phrases, and quotes were grouped together based on similarity of themes regarding decision making, acceptability to the prototype website, and reactions to site design and functionality. Additional codes were created for topics that emerged beyond the initial codes. Similar concepts were summarized and formed into categories, which were evaluated for similarities and differences within and across groups. A grid was constructed to provide an overview summarizing the content of the discussions. Two primary reviewers were utilized to identify these themes, and two secondary reviewers examined the findings to ensure reliability and consensus across reviewers.

## Results

A total of 15 women participated in the study, of which 8 participated in phase 1 and 7 in phase 2. Age of participants ranged from 36 to 78 years, and the average age was 54.8 (SD = 10.93). One third (n = 5) of the participants were African American; the rest were Caucasian. The findings below are separated into three sections highlighting key issues related to the research questions: (1) treatment decision making and finding clinical trials, (2) response to the prototype system for finding breast cancer clinical trials, and (3) site design and functionality.

### Treatment Decision Making and Finding Clinical Trials

The results largely mirrored the findings of previous research that lack of awareness and concerns about experimentation prevented people from considering clinical trial enrollment. Women who had experience with clinical trials, however, were less skeptical about their usefulness and were more open to considering participating in the future. The responses also showed that clinicians could both facilitate and obstruct participation in clinical trials. Clinicians were the main source of information about treatment options, including clinical trials.

All the respondents described having very little time between diagnosis and starting treatment to understand their disease or participate in their treatment decisions. Often their clinicians either recommended a course of action or made decisions as the women were under anesthesia during diagnostic procedures.

Participants’ attitudes toward using a clinical trial matching tool were also influenced by knowledge and clinician support. Although most said they would use the Internet to seek information on clinical trials, most were unfamiliar with clinical trial matching tools. They would be more trusting of an Internet-based matching tool if a clinician recommended it to them, if it was made available in a clinician’s office, or if it was provided by a trusted organization. Women who knew more about clinical trials were less concerned about using an Internet-based clinical trial matching tool than those with less knowledge.

### Perceptions of Prototype Matching System

Phase 1 explored the initial reactions to the system and how it was portrayed to potential users on the home page and in the supporting text for the “About BCT” and the registration process. Table 2 presents the positive and negative reactions to the prototype system among the first phase respondents.

**Table 2:** Positive reactions and concerns to BCT prototype matching system among phase 1 participants

	**Positive Reactions**	**Concerns**
General Reactions	Participants had a favorable reaction to the overall concept and purpose of the site. They considered the idea to have much promise for women interested in finding a trial.	Users were confused by the acronyms (BCT, PHR, etc). Site appeared more focused on the needs of researchers than of breast cancer patients. Many did not appreciate or respond to motivational quotes and artwork unrelated to the site’s purpose, which seemed to add visual clutter but no substance.
Site Entry	Participants were pleased with use of the NCI logo, which improved the site’s legitimacy.	Women wanted more visual cues that the site was focused on breast cancer. They wanted more information about the system and its benefits up front, before having to register. They wanted to see the types of trials available before registering. Participants wanted more information about the demands of trials before registering.
Learning About Clinical Trials	A link to “About Clinical Trials” was used often, especially by women who had little knowledge of clinical trials.	Information provided was for all cancer clinical trials, and women wanted targeted information for breast cancer trials.
Learning About the Site	A link to information about BCT was sought out first by women who had an understanding of clinical trials. Information on the secondary page was considered credible and informative.	The site lacked clear information about the site’s mission and purpose and sponsorship on the home page. Information on the secondary page seemed too long and did not sufficiently address benefits to patients.
Privacy Policy	Participants liked that a privacy policy was available.	Privacy policy seemed long and complex in its wording.
Registration		The emphasis on registration on the home page was off-putting. Many commented on the burden of constantly filling in medical forms. Participants preferred giving personal contact information only after getting matches.
Consent		Consent procedure was seen as long and cumbersome. Consent form seemed to conflict with privacy policy, saying that they risked having information accessed by a nonauthorized source.

Overall, the prototype matching system was seen favorably and as a meaningful innovation for breast cancer patients seeking treatment options. Women noticed information that increased their perceptions of the site’s credibility and read about the sponsors’ concerns about women with breast cancer. However, some women questioned the site’s legitimacy; although not specifically asked to locate information about the site’s sponsorship and mission, the women stated that they wanted to know about sponsorship and were concerned when it was not readily apparent.

When women were confused or felt pushed to register, their reactions became more negative, and they stated more reluctance to using the site. For example, participants had an immediate negative reaction to the prominence of the log-in area on the home page. They stated that they felt uncomfortable with having to register before they knew more about what the system could do for them and the benefits of registration. Feeling driven to register, women stated that the system was developed more for researchers than for breast cancer patients.

The registration process was viewed as cumbersome and reminded the women of filling out extensive medical forms related to their condition. In addition, registration included the need to review and agree to a lengthy consent form, which lengthened the process even more.

As a result of the first phase of triad interviews, several pages of the site were revised. The home page was revised with added information about the benefits of clinical trials, the benefits to using the prototype system, and information about privacy and confidentiality (see Multimedia Appendix). It also used a simple medical image rather than a larger decorative background image. A new page was created to give users who sought information about clinical trials a brief targeted overview of breast cancer clinical trials rather than linking directly to NCI’s website for all clinical trials (ie, not just breast cancer trials), as was done in the original prototype. The page describing the BCT project was also revised to focus on patient benefits.

Participants in the second phase reviewed the revised pages and found them to be clearer and more assuring. Specifically, the users were less skeptical and more open to exploring the system. They seemed more reassured about the system and voiced no reluctance to completing the registration process, unlike the women reacting to the site in phase one.

In the second phase, participants were also asked to review the matches that they could receive after entering all of their information into the system. Women were provided with trial information from NCI’s PDQ Cancer Clinical Trials Registry. The clinical trial records were very confusing and overwhelming to the participants because of the technical medical language. Women were particularly confused about the difference between stage of cancer and phase of clinical trial. Despite this confusion, seeing what trial information looked like helped the participants understand the system much better. Several women wanted to be able to see this type of information earlier in their interaction with the system. Although they liked that the system could tailor the types of trials to their profiles, they wanted to see all possible matches first and then have the ability to drill down themselves.

### Effectiveness of Site Design and Functionality

The third research question of the study was about how the site’s design and functionality affected the match process. These issues were touched on in the first phase of the study and were examined more thoroughly in the second phase as the women had more direct interaction with the site. Table 3 provides a summary of the key design and functionality concerns raised by women in phase 2.

Results demonstrated that users had difficulty using the system on entry, at registration, and when using and completing the PHR. On entry, users were confused about the relevance of the system to their needs. They had trouble locating information that conveyed the site’s credibility, and, when they looked for more information, some of the links sent them to external Web pages without warning. Mostly, the emphasis on registration on the home page made them think they would be forced into joining a fee-based service before they knew what to expect. They were offended by having to provide personal contact information before they received information from the system. As participants proceeded to register on the system, the lack of clear and concise information in the privacy policy and consent form, the lack of clear directions, and poor error handling of the system hampered their progress further.

Once participants entered the personal home page and the PHR-like medical record, they had to orient themselves to new sections with different formats and functionality, and they often had difficulty understanding or using features. The section for describing the various treatments they had received required use of an add/edit/delete link that was not intuitive. Women found that they had trouble moving forward in other sections because of a lack of cues on how to successfully complete the section or what to do next.

Help text was useful but not always available, which was disconcerting for a few questions for which the implications of their answers were unclear. For example, a question asked about one’s willingness to stop current treatment, and women were worried how their answer would affect their relationships with their clinicians or with researchers. Several women stated that they would want to talk to their clinicians before answering certain questions. Others were reluctant to answer questions, especially those asking for personal identifying information, because they wanted to know more about how the information would be used and what to expect in general.

**Table 3:** Design and functionality issues of prototype system for phase 2 participants

Section	Design and Functionality Issues	Illustrative Quotes
Home Page	Emphasis on registration on the home page was off-putting.	“If you have to register even to get past the home page...I’m not that committed to the site yet.” “I think of when I have to [register] as places when I’m buying things.”
	Women wanted design elements that demonstrated credibility and relevance to their needs.	“I'd go for the pink ribbon, because that lets you know it’s a breast cancer site.... You know you’re in the right place.”
	External links to NCI’s clinical trial information page caused users to get lost and even forget about returning to BCT.	“You kind of get over there [on the outside site] and have to think back to ‘What was I originally doing?’.... I totally forgot I was supposed to be looking for clinical trials on this site [bct.org]. I started going deeper and deeper into the NCI site.”
Registration	Users had trouble creating and remembering a username and password due to the confusing process.	“My initial reaction is, why do I have to register, and why do I have to remember another password?”
	No message or cue signaled when registration was successful.	“Is that it? Am I done?” “Where is the part that says I’m successful?”
Personal Home Page	Participants were confused about the purpose of this section compared to the overall home page and the medical record.	“I thought I was going back to the beginning [home].”
	The functionality of the menu items in this section was not clear, so users didn’t know how or why they would use these materials.	“Why do I have an account [My Account]? Is that my personal health history, or my personal home page, or...?”
	The link to start the medical record was difficult to see unless they scrolled down the page—users did not know where to click.	
Uncomfortable Elements	Women were often upset when they were required to give personal information when they wanted to see what matching results would look like.	“If I just want to know where some trials are, why do they have to know really anything about me?”
	“My Cancer” was perceived as asking women to own their disease when they were in a battle to eliminate cancer from their bodies.	“If I leave nothing else with you, [please don’t] call it ‘My Cancer’.... That’s a painful one... to see and have to answer questions on it...just brings you into a reality where you don’t want to be when you’re just trying to get some information.... We try not to take ownership....”
	Users wanted help from a clinician to know how to fill in technical information on diagnosis and treatment, especially when the implications of their responses were unclear.	
Confusing Elements	Users did not recognize the difference between the “Save and Remain on This Page” and the “Save and Go Forward” buttons.	“I'm looking for a way to go back.... It says do not use your browser’s arrow buttons...[but the only available button is save and continue].”
	There was a lack of prompts on how to move forward or retrieve clinical trial matches after filling out the medical record.	“I’d look for something where it says that I've entered this information....” “Where does it tell me to save it? Or has it already saved it?”
Help Mechanisms	“Learn more” buttons were helpful when available. When context-specific help was not available, women struggled to respond.	
	Users had difficulty responding to error messages because of not being able to find the source of the problem or not knowing how to fix it.	“No, [I don’t know why it’s telling me the username is invalid]. Unless my name is just too obvious a name?”
	Too few instructions were available when completing the section about previous cancer treatments. They had difficulty with the add/edit/delete functions.	“It wasn’t clear to me at first.... I’m thinking ‘Surgery, what do they want? Yes, I’ve had it,’ but when I saw Add, Delete, or Edit, how can I edit something that isn’t there?”

## Discussion

This study confirmed many of the findings concerning the design of usable websites and the barriers and motivators to clinical trial participation. It also added knowledge about the clinical trial search process in the context of an Internet-based tool. These findings will be useful to clinical trial researchers and Web professionals designing websites for adults coping with a chronic disease such as cancer.

### Treatment and Clinical Trial Decision Making

In this study, we first examined the treatment decision-making process of individuals diagnosed with breast cancer. The findings confirmed that physicians, especially oncologists, were primary gatekeepers to information on which patients base their treatment plans [9]. Unlike other studies that have looked at specific barriers for cancer patients and breast cancer patients, this study also revealed that time constraints were another significant barrier to participating in clinical trials. Specifically, women newly diagnosed with breast cancer often felt caught in a whirlwind of daunting information with no time between diagnosis and treatment to better understand the disease, much less make treatment decisions. The participants described the breast cancer diagnosis and treatment process as time constrained, emotional, and as focused on following their medical providers’ advice and treatment plans.

The findings also confirmed that women were universally in favor of clinical trials in concept [8,9], but most did not understand the actual process and the demands of participation [11]. The trial description and eligibility criteria were laden with medical terminology, making it difficult for most participants to understand and use the information in order to determine their options and preferences for participation [10]. This study found that time constraints limited the opportunity for patients to become familiar with clinical trials or consider them, as their health care providers were more focused on controlling and eradicating the cancer. These findings suggest that, in order to provide clinical trials as an alternative, health care providers and intermediaries need to be more involved in increasing awareness and knowledge about trials, specifically regarding their role in developing new treatments and the process, benefits, and risks of participation [24].

The study also sought to determine how the idea of a clinical trial search tool fit the needs and expectations of breast cancer patients. Most were unfamiliar with such tools, though a few vaguely remembered seeing online clinical trial search tools on the websites of NCI and the American Cancer Society. Most participants were interested in the concept of a PHR-type clinical trial matching tool. Women who had previous clinical trial experience had fewer concerns than those who had little knowledge of trials. Women said they would be more likely to try a matching service if clinicians recommended it or if the service was made available at the clinician’s office where they could get help using it.

These results confirm previous research showing that cancer patients were willing to use a clinical trial matching tool, particularly if encouraged by health care providers, and that many relied on surrogates to help them [3]. The research suggests that intermediaries—family, clinicians, and clinical staff members—can help women access information on trials by introducing tools like the prototype system that enable them to find trials from which they may benefit.

### Perceptions of Prototype

Our second research question addressed participant reactions to the prototype clinical trial matching system. Previous research found that referrals to clinical trials can be facilitated with technology for the clinician [17] and for patients [3]. Participants in the current study were also favorable to the concept of the prototype and thought it would be helpful to women looking for a breast cancer clinical trial.

This study also provided a unique view of breast cancer patients’ experiences and reactions to the clinical trial search tool that will be valuable to product developers and clinical trial investigators. Looking at the website design recommendations developed by the US Department of Health and Human Services [20] and by the American Association of Retired Persons (AARP) [21], this study confirmed much of the evidence. In general, cues that demonstrated the credibility and legitimacy of the site, like the NCI logo, were well received, and the women searched for such information without being prompted. The women also wanted artwork and graphics that resonated for them—such as a pink ribbon graphic to designate it as a breast cancer site—or images that supported the messages of the site. They did not appreciate graphical elements that seemed unrelated to clinical trials, such as the presentation of artwork done by breast cancer survivors that appeared in the prototype system. These findings confirmed the evidence that relevant graphics were necessary to assure users that a site was credible [20]. Developers need to use pertinent graphical elements and feature the logos of sponsoring organizations with positive standing.

Participants needed more information about how the system could help them before they could develop an interest in it. This finding suggests that developers should provide more explanation about how such an Internet-based tool improves the typical way that women find clinical trials. One way to convey this information would be to make the purpose and benefits of the site easily understandable on the home page. For example, the site could allow women to browse the trials database or could provide a tour of the service. Another way to provide information would be to use second-level Web pages to convey the purpose and functionality of the site, present the benefits of using the site, and make frequently asked questions available. Both of these strategies were put into place for the second phase of the research, and the results received very positive reviews by the participants.

Communicating a website’s purpose clearly on the home page is also a recommendation in the usability evidence base [20]. Although the usability evidence also recommends limiting the prose and length of the home page [20], the participants in this study appeared to favor more information rather than less on the home page as well as the secondary pages.

The findings also revealed that emphasizing and requiring registration on a clinical trial Internet site was a sensitive issue. Prior to being required to register, many participants wanted to understand what they would be asked to do (in terms of information disclosure), what they would get in return via the match process, and what the benefit to them would be. Many women reported being tired of the assumption that they would readily release their personal information to researchers in order to register. While the strategies mentioned above could go a long way toward orienting users, a tutorial or tour may further help potential users to fully understand how the site works, the nature of their involvement, and what the results could be. Allowing users to browse trials before registering would also enable them to learn about the types of trials available and what they could expect to get out of engaging in the process.

### Design and Functionality Issues

The third research question examined how the website’s design and functionality affected the clinical trial match process. The current study confirmed findings in the usability literature about specific design features and functionality.

Usability research recommends that critical information be placed high in the hierarchy of the website and that information be organized clearly [20]. In the current study, the lack of knowledge about clinical trials was very common, and women sought information about clinical trails first. These findings support providing a link to site-specific clinical trial information (“About Clinical Trials”) first among the options in the menu structure to help explain clinical trials upfront.

The study confirmed the usability recommendation to liberally use descriptive headings to help users conceptually relate to the content [20]. Participants, for example, had strong negative reactions to the label “My Cancer,” believing the site asked them to “own” their cancer when they were trying to rid themselves of it. Therefore, these results also indicate that developers should pay careful attention to creating headings that are acceptable and sensitive to the target audience’s situation.

The study showed that the opportunities for confusing potential users of this system proved to be great. The topic of clinical trials was difficult to present, in general, which is consistent with previous research [11]. Here, the women also found that the terminology related to breast cancer diagnosis and treatment and the privacy policy added to the overall complexity. The use of the Internet and informatics terms and acronyms like PHR further confused and overwhelmed the users trying to navigate the site. Limiting the use of acronyms and medical jargon and using terms that are more recognizable to the lay public are important in the content development of usable websites [20]. These findings support the placement of context-specific help text to address user questions at the point at which they are having trouble and to ensure ease of navigation. Users can also be prompted and guided on how to get assistance from their medical providers so they get the most out of the system.

The study also confirmed the recommendation to provide assistance to users, especially novices and first-time users [20]. Many breast cancer patients are likely to be older women, and older adults tend to have less experience and expertise with computers and the Internet [21]. As a result, participants often required additional help with registration tasks such as selecting a username and password. They were also more likely to require assistance when the system’s navigational cues and input strategies varied because they did not know what they needed to do to enter information or move on in the program [21]. Therefore, the developers of such sites should strive to give clear directions and specific examples to facilitate use among the older adult target audience.

Previous usability studies have indicated that developers should indicate when a link will move users to a different location on the same page, a new page, or a different website [20]. The current study confirmed that external links were problematic. Specifically, users who clicked on the “About Clinical Trials” were confused when they went to the NCI website and had trouble getting back to the prototype site. Some even forgot about the prototype website and became engrossed in the information about clinical trials on the NCI site.

The evidence from usability research has indicated that action sequences should be developed so they are easily understood [20]. The tasks involved in the prototype system were complex in nature. This research suggests that users would benefit from revised instructions and additional prompts, especially when completing their PHR. For example, the section where users recorded medical treatments (“My Treatment”) could be enhanced with better design or directions on how to add multiple entries (eg, if they had two lumpectomies in the same breast but at different times) or to delete entries. Once a task was completed, the system could also send a message to let participants know whether or not they successfully completed the task, such as registering on the site or completing the health record. If they did not successfully complete the task, the system should provide instructions of what they still need to do in order to be matched to clinical trials.

### Conclusions and Recommendations

This study built upon previous research on clinical trial recruitment and usability, finding that even novice and older adult users were open to an Internet-based clinical trial matching system. In terms of breast cancer diagnosis and treatment, the system must also fit into the context of an emotionally charged, time-limited situation. Such a system must also fulfill expectations for credibility and benefit as well as privacy so that women feel confident and assured in their participation.

One limitation of the study was the use of a qualitative research design, which means that we cannot generalize the results to other breast cancer patients. Future research is needed that provides quantitative data and uses randomization. Another limitation was that the participants were not actually in the process of searching for a cancer clinical trial—they were only responding to a hypothetical situation. Therefore, their decision-making processes might have been different if they were truly looking for a clinical trial. Recruiting breast cancer patients for this study was challenging in general, but future research will be needed with women who are in the process of considering clinical trials. Lastly, the number of participants might be considered small by some researchers. Although the study may have benefited from increasing the sample size, user-centered research shows that as few as six people is sufficient.

The research confirmed one of the core tenets of usability engineering, that one must use an iterative design approach [20]. Checking with users and observing them with the site ensures relevance and usefulness of the resulting product. Although user-centered research takes additional time and effort, it saves time and money in the long run by identifying problems early.

Another primary approach in usability engineering is evaluating websites both before and after making changes. This was a first step in our research process. Future research is needed to confirm that changes made to the prototype system based on the user-centered research improve its usefulness and increase satisfaction among the target audience [20]. Qualitative research can be used to further examine user reactions. However, research could also be done to compare how easily and effectively users can navigate and use the prototype and modified websites. Most importantly, outcome research is needed to determine whether such tools actually result in more and better matches. Translational research would also enable exploration of how clinical trial matching systems could best be integrated into different settings, especially medical facilities and the home.

## Multimedia Appendix

Screenshots of the Web site (ppt)
					

## Abbreviations

BCT: BreastCancerTrials.org
NCI: National Cancer Institute
PHR: personal health record
