# Supplementary figures and images for: User-Centered Research on Breast Cancer Patient Needs and Preferences of an Internet-Based Clinical Trial Matching System
Source: J Med Internet Res. 2007 May 15;9(2):e13. doi: 10.2196/jmir.9.2.e13 (PMC1874719; doi:10.2196/jmir.9.2.e13)

## Slide 1
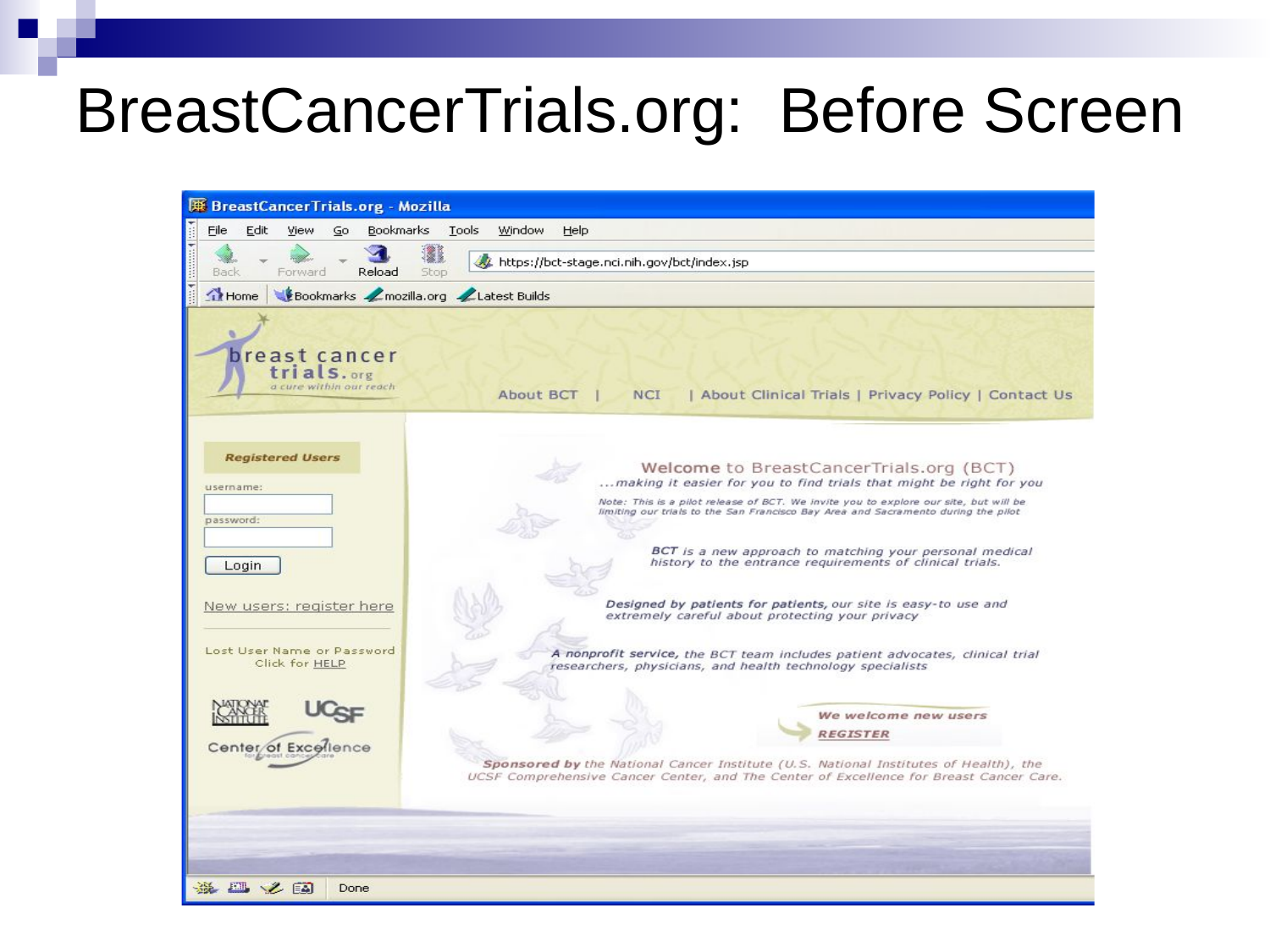

# BreastCancerTrials.org: Before Screen

## Slide 2
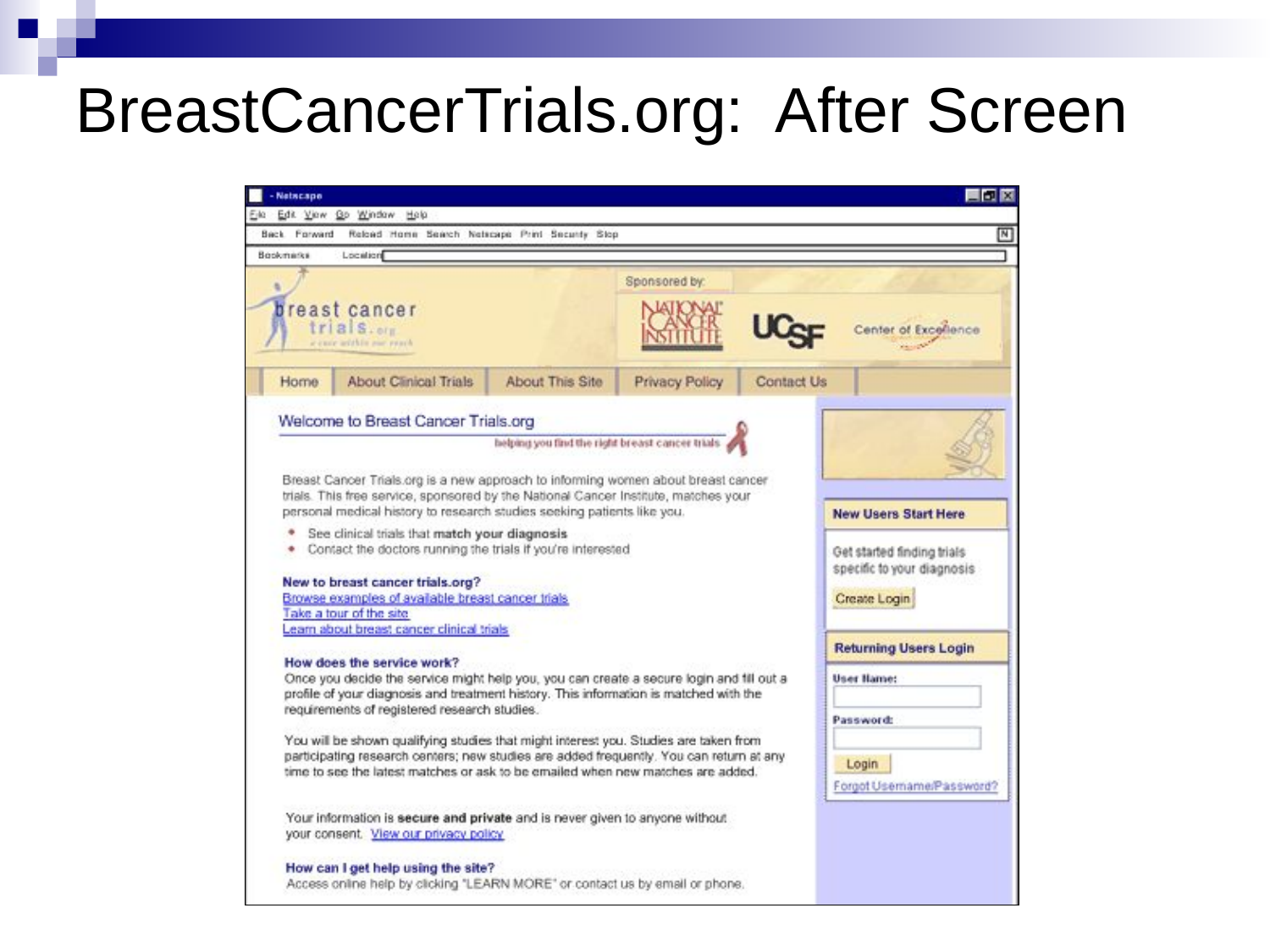

# BreastCancerTrials.org: After Screen

Supplement: Supplementary file 1 [file jmir_v9i2e13_app1.ppt]
